# The complete chloroplast genome sequence of wild oat, *Avena sterilis* L. (Poaceae) and its phylogeny

**DOI:** 10.1080/23802359.2018.1444518

**Published:** 2018-03-05

**Authors:** Raveendar Sebastin, Kyung Jun Lee, Myoung-Jae Shin, Gyu-Taek Cho, Kyung-Ho Ma, Jung-Ro Lee, Gi-An Lee, Jong-Wook Chung

**Affiliations:** a National Agrobiodiversity Center, National Institute of Agricultural Sciences, RDA, Jeonju-Si, Republic of Korea;; b Department of Industrial Plant Science and Technology, Chungbuk National University, Cheongju, Republic of Korea

**Keywords:** *Avena sterilis*, chloroplast, illumina sequencing, wild oat

## Abstract

Wild oat, *Avena sterilis* L. is a stout broad-leaved annual grass resembling cultivated oats in general appearance. In this study, we sequenced the complete chloroplast (cp) genome sequence of *A. sterilis* for the first time to investigate their phylogenetic relationship in the family Poaceae. The complete cp genome sequence is 135,887 bp in length with 38.5% overall GC content and exhibits a typical quadripartite structure comprising one pair of inverted repeats (21,603 bp) separated by a small single-copy region (12,575 bp) and a large single-copy region (80,106). The cp genome encodes 111 unique genes, 76 of which are protein-coding genes, four rRNA genes, 30 tRNA genes, and 18 duplicated genes in the inverted repeat region. The phylogenetic analysis indicated *A. sterilis* closely clustered with the cultivated oat, *A. sativa* L.

## Introduction

Wild oat (*Avena sterilis* L.) is an autogamous grass and has a global geographical distribution (Armengot et al. 2008). Under the view of genetic erosion, a number of wild *Avena* L. species were used as donors of valuable characters (Loskutov 1998). Understanding the relationships between *Avena* species is vital for efficient transfer of exotic genes to cultivar germplasm. Chloroplast genome sequencing of wild species has been extensively applied to understand the plant genetic diversity and evolution (Liu et al. 2016; Park 2016, 2017; Tsuruta et al. 2017). Chloroplast genome sequence of a cultivated oat *Avena sativa* L. was reported (Saarela et al. 2015). In this study, we report the chloroplast genome sequence of *Avena sterilis* L. to find its internal relationships within the family Poaceae.

Wild oat seeds (Accession No. IT189857) were obtained from the National Agrobiodiversity Center, Republic of Korea. Seeds were germinated and fresh leaves were collected from 40-day-old seedlings. Total genomic DNA was extracted to build up genomic library and sequenced with pair-end (2 × 300 bp) by MiSeq instrument at LabGenomics (http://www.Lab.genomics.com/kor/). A total of 4,362,380 raw read and 3,061,845 clean reads were obtained, and mapped with the reference cp genome, *A. sativa* L. (GenBank accession KM974733), which contains 48,939 aligned reads with about an average 79 × coverage. Contig alignment and scaffolding based on paired-end data resulted in a complete circular cp genome. DOGMA (http://dogma.ccbb.utexas.edu/) software was used for annotation of protein coding genes in the cp genome and manually inspected to predict transfer RNA (tRNA) and ribosomal RNA (rRNA) genes.

The total length of the chloroplast genome is 135,887 bp, with 38.5% overall GC content (NCBI accession number KX756180). A pair of IRs (inverted repeats) of 21,603 bp was separated by a small single copy (SSC) region of 12,575 bp and a large single copy (LSC) region of 80,106 bp. The chloroplast genome harbors 111 known genes, including 76 protein-coding genes, four ribosomal RNA genes, and 30 tRNA genes. A total of 18 genes were duplicated in the inverted repeat regions, eight genes, and two tRNA genes contain one intron, while ycf3 have two introns.

**Figure 1.**
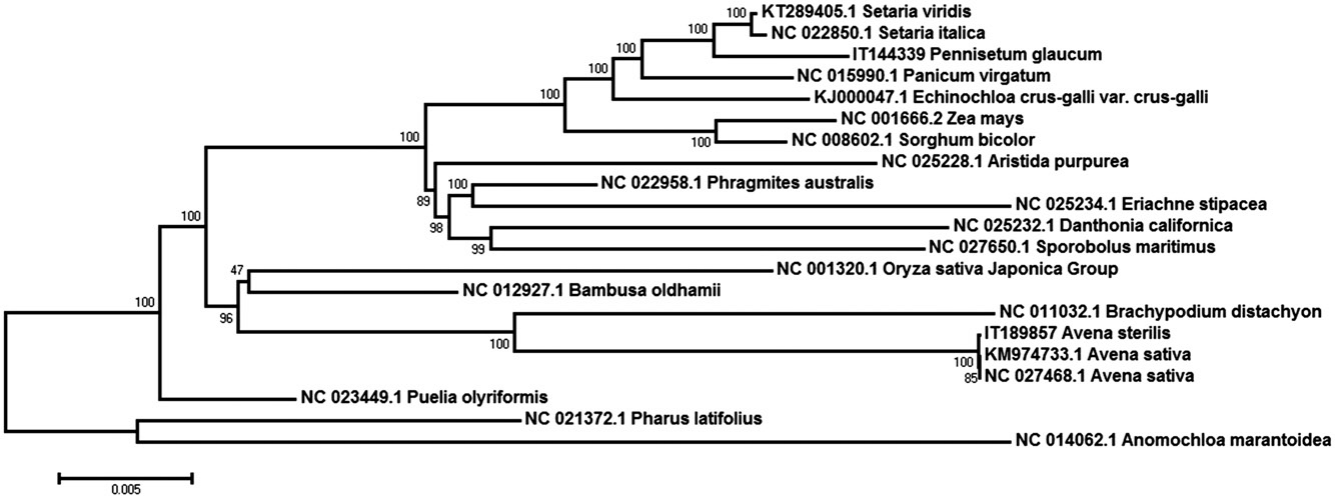
Maximum likelihood (ML) phylogeny of wild oat, *A. sterilis* and other related species in Poaceae based on complete chloroplast genome. The complete chloroplast genome is downloaded from NCBI database and the phylogenetic tree is constructed by MEGA6 software.

To analyse the phylogenetic relationships, the published chloroplast genome sequences from Poaceae species were downloaded from the NCBI database (Figure 1). Whole genome sequence were aligned by MAFFT v7.304 (Katoh and Standley 2016) and MEGA6 (Tamura et al. 2013) software was used to construct a maximum likelihood (ML) tree with 1000 bootstrap replicates. Phylogenetic analysis indicated *A. sterilis* clustered together with *A. sativa* L. and clustered with *brachypodium distachyon* belonging to the sub-family Pooideae. Phylogenomic analyses of Poaceae cp genomes including this newly sequenced genome resulted in a highly resolved phylogeny. The cp genome of *A. sterilis* will provide as useful resources for germplasm collection, conservation, and utilization.

## References

[CIT0001] Armengot L, L, L, A, Sans FX. 2008. L, JoséMaría L, Chamorro L, Romero A, Sans FX. 2008 The effect of Avena sterilis L. invasion on weed abundance and diversity in conventional and organic cereal fields in the Mediterranean region. Poster at: Cultivating the Future Based on Science: 2nd Conference of the International Society of Organic Agriculture Research ISOFAR, Modena, Italy, June 18-20.

[CIT0002] Katoh K, Standley DM. 2016 A simple method to control over-alignment in the MAFFT multiple sequence alignment program. Bioinformatics. 32:1933–1942. 27153688 10.1093/bioinformatics/btw108 PMC4920119

[CIT0003] Liu F, Tembrock LR, Sun C, Han G, Guo C, Wu Z. 2016 The complete plastid genome of the wild rice species *Oryza brachyantha* (Poaceae). Mitochondrial DNA Part B. 1:218–219. 10.1080/23802359.2016.1155093 PMC7871827 33644346

[CIT0004] Loskutov IG. 1998 The collection of wild oat species of CIS as a source of diversity in agricultural traits. Genet Resour Crop Evol. 45:291–295. [English].

[CIT0005] Park T-H. 2016 The complete chloroplast genome sequence of potato wild relative species, *Solanum nigrum*. Mitochondrial DNA Part B. 1:858–859. 10.1080/23802359.2016.1250133 PMC7800014 33473656

[CIT0006] Park T-H. 2017 The complete chloroplast genome of *Solanum berthaultii*, one of the potato wild relative species. Mitochondrial DNA Part B. 2:88–89. 10.1080/23802359.2017.1285213 PMC7800855 33473725

[CIT0007] Saarela JM, Wysocki WP, Barrett CF, Soreng RJ, Davis JI, Clark LG, Kelchner SA, Pires JC, Edger PP, Mayfield DR, et al 2015 Plastid phylogenomics of the cool-season grass subfamily: clarification of relationships among early-diverging tribes. AoB Plants. 7. doi: 10.1093/aobpla/plv046 10.1093/aobpla/plv046 PMC4480051 25940204

[CIT0008] Tamura K, Stecher G, Peterson D, Filipski A, Kumar S. 2013 MEGA6: molecular evolutionary genetics analysis version 6.0. Mol Biol Evol. 30:2725–2729. 24132122 10.1093/molbev/mst197 PMC3840312

[CIT0009] Tsuruta S-i, Ebina M, Kobayashi M, Takahashi W. 2017 Complete chloroplast genomes of erianthus arundinaceus and miscanthus sinensis: comparative genomics and evolution of the saccharum complex. PLoS One. 12:e0169992. 28125648 10.1371/journal.pone.0169992 PMC5268433

